# Comparison of premenstrual symptoms, psychological well-being, and nutritional status between Palestinian women with and without polycystic ovarian syndrome: a case-control study

**DOI:** 10.1186/s12905-024-03210-z

**Published:** 2024-06-21

**Authors:** Marwa Almahareeq, May Hamdan, Divya Vanoh, Nuha Shawarb, Jana Herbawi, Eman Shawar, Raneen Al-wohoush, Manar Mohtaseb, Manal Badrasawi

**Affiliations:** 1 Faculty of Medicine and health sciences, An-Najah National University, Nablus, Palestine; 2 Department of Health professions, Program of Healthy and Therapeutic Nutrition, Faculty of Medicine, Palestine Polytechnic University, Hebron, Palestine; 3 Program of Nutrition and Dietetics, School of Health Sciences, Universiti Sains Malaysia, Health Campus, Kubang Kerian, Malaysia; 4 Department of Nutrition and Food Technology, Faculty of Agriculture and Veterinary Medicine, An-Najah National University, Tulkarm, PO. Box 7, West Bank Palestine

**Keywords:** Polycystic ovarian syndrome, Premenstrual syndrome, Mental health, Case-control, Predictors

## Abstract

**Background:**

Polycystic ovarian syndrome (PCOS) is a widely seen reproductive and endocrinological disorder. PCOS can exert substantial effects on many aspects of an individual’s life, including reproductive health and psychological well-being. The objective of this study was to assess the nutritional status, premenstrual syndrome, and mental health of women affected by PCOS in comparison to women without PCOS.

**Methodology:**

A case-control observational study in Palestine included 100 PCOS patients and 200 healthy women. The collected data included socio-demographic information, medical history, premenstrual syndrome, mental health, nutritional status, and lifestyle. Anthropometric measurement and the Mediterranean Diet Adherence Screener (MEDAS) were used to evaluate the nutritional status. The General Health Questionnaire (12-GHQ) was used to evaluate the state of mental health. Premenstrual syndrome (PMS) severity was evaluated using a validated Arabic premenstrual syndrome questionnaire.

**Results:**

The study’s findings indicated that there was a statistically significant increase in the three dimensions of PMS among participants with PCOS, *p* < 0.05. Similarly, PCOS patients demonstrated elevated ratings across all aspects of mental health, *p* < 0.05. In terms of the other variables, it has been observed that PCOS patients have a notably greater prevalence of perceived sleep disturbances and decreased adherence to the Mediterranean diet. Regression analysis revealed that PCOS is associated with mental health problems indicated by a higher GHQ score (OR: 1.09; 95% CI: 1.03; 1.16, *p* < 0.05), lower adherence to the MD diet (OR: 0.86; 95% CI: 0.76; 0.98, *p* < 0.05), and pre-menstrual syndrome, especially the physical symptoms (OR: 1.06; 95% CI: 1.003; 1.12, *p* < 0.05) after adjusting for age, smoking, waist-hip ratio, and body mass index (BMI).

**Conclusion:**

The study has linked polycystic ovary syndrome to negative mental health outcomes and an increased severity of premenstrual syndrome (PMS). Additional investigation is required in order to establish a causal association between polycystic ovary syndrome (PCOS) and lifestyle behaviors within the Palestinian population. Intervention and instructional studies are necessary to investigate the efficacy of management strategies in alleviating the effects of polycystic ovary syndrome (PCOS) on both physical and mental well-being.

## Background

Polycystic ovary syndrome (PCOS) is a prevalent reproductive and endocrinologic disorder that affects the female population [1]. In the last decade, the proportion of women with PCOS has increased, with a pooled mean prevalence of 21.27% using different diagnostic criteria based on a recent systematic review [2]. PCOS is defined by a combination of signs and symptoms of androgen excess and ovarian dysfunction in the absence of other specific diagnoses [3]. Researchers are still actively researching the exact etiology and pathogenesis of PCOS, and they have postulated multiple hypotheses, ranging from genetic susceptibility to environmental exposure, both in utero and in postnatal life [4]. The World Health Organization’s (WHO) data suggests that approximately 116 million women (3.4%) are affected by PCOS globally [5] and 7.3% in Palestine [6]. PCOS is characterized by polycystic ovarian morphology (PCOM), ovulatory dysfunction, menstrual disorders, reproductive problems, infertility, oligomenorrhea, hyperandrogenism, and some clinical manifestations of alopecia, acne, oily skin, and hirsutism [7]. Moreover, several complications, including insulin resistance (IR) [8], type 2 diabetes (T2DM) [9], cardiovascular disease (CVD) endometrial cancer [7], mental and behavioral disorders (e.g., anxiety, depression, and lack of self-confidence), dyslipidemia [9], metabolic syndrome [10], and specifically obesity, are associated with untreated PCOS [9]. Multiple strategies, tailored to the individual patient’s condition and status, comprise the management method for polycystic ovary syndrome (PCOS). One of these strategies is the use of oral contraceptives to reduce testosterone synthesis [11].

Women with PCOS who are classified as obese or overweight may potentially experience positive effects from the use of metformin. In the case of excessive hair growth, the administration of drugs that inhibit androgens, such as spironolactone, is beneficial. However, women who wish to conceive should avoid these medications, as they may pose risks to the developing fetus [12]. Nutrition plays a major role in improving related medical issues. Nutrition and lifestyle interventions, including weight loss, dietary modifications, and exercise, have documented effectiveness in PCOS management and should be given top priority [11]. The implementation of lifestyle improvements, such as engaging in regular exercise and achieving weight loss, had a positive impact on insulin resistance and ovulatory function in women diagnosed with polycystic ovary syndrome (PCOS) [1]. The utilization of low-glycemic-index carbohydrates has been observed to yield advantageous outcomes in the management of clinical symptoms associated with polycystic ovary syndrome (PCOS), such as insulin resistance, lipid profile, and ovulation-related outcomes [5]. In another study, the modified hypocaloric diet (MHCD) lowered testosterone levels and low-density lipoprotein cholesterol (LDL-C) significantly after a 12-week intervention period, whereas FSH, LH, and blood lipids remained unaltered [13]. The impact of the Mediterranean diet and lifestyle on alleviating symptoms associated with polycystic ovary syndrome (PCOS) has been well documented. The Mediterranean region, encompassing Palestine, is recognized for its adherence to a healthy lifestyle, marked by a high level of physical activity and a preference for nutritious dietary selections and culinary methods. These factors contribute to promoting a healthy diet for those with Polycystic Ovary Syndrome (PCOS) [9].

The clinical manifestations of premenstrual syndrome (PMS) encompass a range of physical symptoms, including but not limited to edema, breast tenderness, headaches, heightened appetite, and heart palpitations. Additionally, PMS is associated with various behavioral and psychological symptoms, such as depression, irritability, fatigue, aggression, suicidal ideation, impaired concentration, mood fluctuations, and social isolation. Premenstrual syndrome (PMS) is a prevalent, untreated ailment that poses a significant public health concern among women of reproductive age. Research has revealed that this condition negatively impacts mental well-being, overall quality of life, and academic performance. A comprehensive study and meta-analysis revealed a global prevalence of premenstrual syndrome (PMS) of 48%. Furthermore, research conducted in Iran has indicated that the prevalence of premenstrual syndrome (PMS) ranges from 30 to 99.5% [14]. The etiology of premenstrual syndrome (PMS) is currently not fully understood. However, it is believed that cyclical ovarian activity and the impact of estradiol and progesterone on the neurotransmitters serotonin and gamma-aminobutyric acid (GABA) play significant roles in its development [15]. Research has revealed that the fluctuation in hormone levels associated with PCOS and PMS exacerbates the symptoms of PMS and causes temporary mood swings [16]. Additionally, women with PCOS may also experience PMS [17].

Women diagnosed with PCOS experience prolonged anovulation or oligo-ovulation. As a result, PCOS is characterized by a significant deficiency of progesterone and its derivatives. The hypothesis suggests that a shortage of progesterone may potentially exacerbate the severity of premenstrual syndrome symptoms in women with PCOS [18]. However, it is crucial to acknowledge the scarcity of empirical studies exploring this relationship. Furthermore, there is a lack of research on the severity of premenstrual syndrome (PMS) in women with PCOS versus women without PCOS. To our knowledge, there is no published research on the nutritional status, lifestyle, PMS symptoms, or mental health of Palestinian women with PCOS. The study aims to evaluate the impact of polycystic ovarian syndrome (PCOS) on the health of Palestinian women, specifically examining weight changes, lifestyle factors, reproductive health, mental wellness, premenstrual symptoms, and adherence to dietary guidelines. The findings will provide guidance for interventions aimed at enhancing the quality of life for people with PCOS. These interventions may include nutrition education and exercise programs. Additionally, the findings highlight the significance of identifying PCOS biomarkers to improve the management of the condition.

## Methods

### Study design, setting, and population

This case-control study was carried out among 300 women (100 women with PCOS and 200 women as controls) aged 18 or older living in Hebron City. The study protocol has been approved by the internal review board for research ethics at Palestine Polytechnic University. Informed consent had been obtained from all the participants before data collection. For the PCOS case recruitment, four well-known gynecological clinics were officially contacted by the research team, briefed about the study, and asked if they were willing to help identify the participants with a confirmed PCOS diagnosis. Patients who were previously diagnosed with PCOS and visited the clinic for follow-up were asked by their gynecologist if they agreed to join the study. Participants who verbally agreed and met the inclusion criteria were asked to sign the consent form. The diagnosis of PCOS was done by the gynecologists based on Rotterdam diagnosis criteria: the presence of two of three of the following criteria: oligoanovulation, hyperandrogenism, and polycystic ovaries (≥ 12 follicles measuring 2–9 mm in diameter and/or an ovarian volume > 10 mL in at least one ovary) [19]. The inclusion criteria for the cases were women with a confirmed diagnosis of PCOS for more than three months, age 18 years and older, who agreed to join the study. After selecting the PCOS patients, healthy controls, aged 18 years and older, were invited to join the study from the neighboring clinics who came for medical consultation for reasons other than gynecological problems. In order to confirm that the controls do not have PCOS, they were required to meet two specific criteria: having a normal and regular menstrual period that falls within the range of 26–33 days, and having a recent pelvic ultrasound taken within the past three months that shows no abnormalities or concerning findings, indicating the absence of any signs of disease or abnormality. The matching was done depending on age and marital status. The exclusion criteria for both groups (cases and control) included a woman who suffered from congenital adrenal hyperplasia, thyroid dysfunction, hyperprolactinemia, or any diseases whose symptoms are similar to or overlap with PCOS, pregnant women, or women who did not complete the questionnaire.

### Sample size calculation

The sample size was calculated using the G Power software version 3.1.7.9 for sample size calculation for two independent groups. The mean and standard deviation from the previous study were used to calculate the required sample [20]; the effect size was considered 0.8; the level of significance was alpha 0.05; and the allocation ratio was 1:2. The required sample size was 100 PCOS patients and 200 healthy controls.

### Data collection and study instruments

The data collection started in May 2022 and ended in August 2022. The data were gathered through the utilization of a pre-established questionnaire that encompassed many sections. These sections included sociodemographic information, medical history, lifestyle factors, PCOS-related data (specifically for the cases), reproductive health-related parameters, mental health indicators, premenstrual syndrome evaluation, and assessment of nutritional status.

The socio-demographic data included: age, marital status, living area, educational level, work status, and income. The lifestyle data included smoking history, exercise, and perceived sleeping problems. Medical history: the presence of chronic diseases other than PCOS, previous surgical procedures, and supplement use. Reproductive data for both PCOS cases and the control included: the number of pregnancies, the number of miscarriages for married participants, the age of menarche, and the menstruation period. PCOS-related data collected from the cases only included the age of diagnosis, diagnosis method, duration of the disease, and the management protocol. Nutritional status assessment was done using anthropometry measurements: weight, height, waist circumference, hip circumference, and waist-hip ratio. All the measurements were done following the measuring protocol (Lee and Neiman). Dietary pattern was assessed using a validated 14-item Questionnaire of the Mediterranean Diet (MEDAS) to assess adherence to the Mediterranean diet as a healthy and recommended diet in Palestine, a Mediterranean country [21]. To classify the degree of adherence to the Mediterranean pattern, a more generalized scale was used: scores less than 4 indicate “low adherence,” scores ranging from 4 to 6 indicate “intermediate adherence,” and scores more than 6 indicate “high adherence” [22].

Mental health was assessed using the validated Arabic version of the General Health Questionnaire (12-GHQ) [23]. The General Health Questionnaire is a widely used tool to assess mental health in the general population. The total scores of GHQ-12 range from 0 to 36; higher scores indicate worse mental health. An overall score of more than 15 indicates the presence of mental health issues [23].

The presence and severity of premenstrual syndrome PMS were assessed using the Arabic Premenstrual Syndrome Scale (APMSS), developed by Algahtani and Jahrami (2014). APMSS consists of 23 items; each question has a 5-point Likert scale (never, sometimes, often, always, and severely), and each answer has a given score. PMS symptoms’ severity among the sample was calculated by the mean of each category’s question score [24].

### Statistical analysis

Data were coded and analyzed using the Statistical Package for Social Sciences (SPSS) software version 23. Descriptive analysis, including the means and standard deviation, was used to analyze the continuous variables, and categorical variables were described in percentages. The inferential statistical tests were used according to the variables and the number of groups. Univariate analysis was done using Chi-square tests and the mean difference. Multivariate analysis was done using binary logistic regression to determine the association between PCOS and various variables selected from the univariate analysis or obtained from previous literature. The model has been adjusted for age, smoking status, body mass index (BMI), and waist-hip ratio (WHR). The significance value was set at *p* < 0.05.

## Results

### Participants characteristics

Table 1 shows participants’ characteristics—socio-demographic and lifestyle—displayed in numbers and percentages. Significant associations were seen between PCOS and perceived sleep problems, in comparison to the control group, among the variables that were reported in Table 1.

**Table 1:** Sociodemographic, medical history, and lifestyle characteristics of the participants according to the groups presented in n (%)

Variables		PCOS Case *n* = 100	Control *n* = 200	*P*-value
**Sociodemographic**				
Living location	City	54 (54)	167 (83.5)	0.11
	Village + camp	46 (46)	33 (16.5)	
Educational level	School level	34 (34)	42 (21)	0.17
	Bachelor’s degree/ diploma,	66 (66)	158 (79)	
Family income in Shekel currency	Less than 1500	5 (5)	8 (4)	0.721
	From 1500- <3000	37 (37)	65 (32.5)	
	From 3000-<5000	36 (36)	72 (36)	
	Above 5000	22 (22)	55 (27.5)	
Working status	Working	21 (21)	60 (30)	0.192
	No working	79 (79)	140 (70)	
**Medical history**				
Chronic diseases other than PCOS	Yes (%)	9 (9)	23 (11.5)	
Previous surgical operation		18 (18)	47 (23.5)	
**Lifestyle**				
Smoking	Regular	11 (11)	23 (11.5)	0.956
	Irregular	81 (81)	159 (79.5)	
	Non-smoker	8 (8)	18 (9)	
Doing exercise	Yes, regularly^1^	7 (7)	9 (4.5)	0.071
	Yes, irregular^2^	38 (38)	54 (27)	
	No	55 (55)	137 (68.5)	
Perceived Sleep problem	Yes, always	28 (28)	32 (16)	0.000*
	Sometimes	36 (36)	104 (52)	
	Never	36 (36)	64 (32)	

^1^regular: participation in an exercise program, accompanied by a trained professional

^2^irregular: without regular professional advice, performed at home [25]

### Reproductive health and PCOS

Table 2 shows the differences between the PCOS cases and the control in pregnancy-related data. Based on our data, 43 (61.4%) of women with PCOS struggled with delayed pregnancy; 18.9% needed infertility treatment; 12.1% needed assisted reproductive technology (ART); and 3.5% needed both. While 86.1% of the control group were able to get pregnant naturally without any intervention. There wasn’t a difference between the two groups regarding the number of pregnancies (case: 3.3 ± 2.5, control: 3.6 ± 2.1, *p* > 0.05 using an independent sample t-test). There was no significant difference between the two groups regarding having a miscarriage (case: 1.70 ± 1.51, control: 1.51 ± 0.73, *p* > 0.05 using an independent sample t-test).

**Table 2:** the difference between PCOS cases and controls according to pregnancy-related data

Variables		Case (*n*%) *N* = 100	Control (n%) *N* = 200	*P*- value
History of pregnancy	Yes	49 (68)	80 (86.9)	0.003*
History of miscarriage	Yes	22 (30.9)	29 (31.5)	0.540
Delay in pregnancy (> 1 year)	Yes	43 (61.4)	20 (21.9)	0.000*
pregnancy method	Normal Pregnancy	38 (65.5)	68 (86.1)	0.028*
	Medical treatment	11 (18.9)	8 (10.2)	
	ART	7 (12.1)	2 (2.5)	
	Both (medical treatment and ART)	2 (3.5)	1 (1.2)	

*ART* Assisted reproductive technology

### Premenstrual syndrome (PMS) symptoms and mental health status

The findings of the study indicate that patients with polycystic ovary syndrome (PCOS) experience considerably higher premenstrual symptoms, as evidenced by their subdomain scores, compared to the control group. This suggests that the severity of premenstrual syndrome (PMS) symptoms is greater among those with PCOS. Regarding mental health, based on the General Health Questionnaire (GHQ), there was a significant relationship between PCOS and mental health issues (*P* < 0.001), as shown in Table 3. When categorizing participants’ mental health as either normal or having a mental health issue, a significantly higher percentage of mental health issues were observed among participants with PCOS (43%) compared to those without (17.5%), *p* < 0.01 using the Chi-square test.

**Table 3:** The difference between case and control according to PMS symptoms and mental health—PCOS relationship

PMS symptoms and mental health	Case (*n* = 100) Mean ± sd	Control (*n* = 200) Mean ± sd	*P*-value
Psychological Symptoms	22.7 ± 8.2	18.44 ± 9.4	0.001*
Physical symptoms	17.6 ± 6.6	13.7 ± 6.5	0.001*
Functional impairment	3.6 ± 2.7	2.6 ± 2.7	0.004*
Mental health- total	15.1 ± 5.4	11.6 ± 4.9	0.001*
Mental health- Anxiety and depression	6.1 ± 2.7	2.5 ± 2.5	0.001*
Mental health- Social dysfunction	7.6 ± 2.4	6.3 ± 2.4	0.001*
Mental health- loss of confidence	1.4 ± 1.9	0.7 ± 1.1	0.001*

*Significant *p*<0.05 using independent sample t-test, *PMS* Premenstrual syndrome

### Nutrition-related variables

A total of 54 (54%) females with PCOS were either obese or overweight compared to 105 (52.5%) overweight and obese females in the control group, *p* > 0.05 using the chi-square test. The association between PCOS and eating habit satisfaction and weight satisfaction intake did not reach a statistically significant level. On the other hand, there was a significant association between perceived changes in weight. Perceived weight changes were reported to be higher among PCOS women (73%) as compared to the control group (61.5%), *p* < 0.05. Meanwhile, the PCOS group also demonstrated an increase in supplement intake (44%) as compared to the control group, which only reported 22.5% (*p* < 0.05). Interestingly, a lower adherence to the Mediterranean diet (MD) was reported among PCOS individuals compared to the control group, as shown in Table 4. Even though both groups show high adherence to MD, as indicated by scores greater than 6. However, there was no statistical relationship between PCOS and BMI or WHR.

**Table 4:** The differences in nutrition-related variables between the PCOS cases and the control

Nutrition-related variables	Case (*n* = 100) Mean ± sd	Control (*n* = 200) Mean ± sd	*P* value
Body mass index	25.9 ± 5.1	25.7 ± 4.9	0.709
WHR	0.82 ± 0.07	0.82 ± 0.06	0.689
Adherence to Mediterranean diet	7.8 ± 1.9	8.6 ± 2.3	0.003*

*Significant *p*<0.05 using independent sample t-test, *WHR* Waist-to-hip Ratio

### Multivariate analysis

Among the factors associated with PCOS are the GHQ score, PMS physical score, and adherence to the Mediterranean diet (MD). Increasing GHQ score is associated with increased odds of PCOS by 1.09 (95% CI: 1.03; 1.16, *p* < 0.05). Similarly, higher scores in the PMS physical domain are also associated with an increased risk of PCOS (OR: 1.06; 95% CI: 1.003; 1.12, *p* < 0.05). In addition, moderate and high adherence to the MD diet is associated with 0.86 lower odds of PCOS (95% CI: 0.76; 0.98, *p* < 0.05) Table 5.

**Table 5:** Factors Associated with PCOS

Parameters	Estimate	Standard Error	Odd Ratio	95% CI	*P*-value
Chronic disease	0.69	0.62	1.99	0.59;6.71	0.500
OTC Drugs	-0.34	0.54	0.72	0.25;2.06	0.530
Sleep difficulty	0.21	0.29	1.23	0.70;2.16	0.460
Waist circumference	-0.33	0.22	0.72	0.47;1.09	0.126
Hip circumference	0.28	0.19	1.32	0.92;1.89	0.136
GHQ Score	0.09	0.03	1.09	1.03;1.16	0.002*
PMS psychological	-0.001	0.02	0.99	0.95;1.04	0.950
PMS physical	0.06	0.03	1.06	1.003;1.12	0.039*
PMS behavioral	0.02	0.06	1.02	0.91;1.14	0.706
Weight changes	0.24	0.31	1.51	0.54;4.24	0.440
MD score	-0.15	0.065	0.86	0.76;0.98	0.018*

*Significant at *p* < 0.05

Dependent variable: PCOS status (0: no PCOS, 1: PCOS). Model adjusted for age, body mass index, smoking status, and waist-hip ratio

*Abbreviation*: *OTC* Over-the-counter drugs, *CI* confidence interval, *GHQ* general health questionnaire, *PMS* pre-menstrual syndrome, *MD* Mediterranean diet

## Discussion

To our knowledge, this is the first case-control study comparing premenstrual syndrome, nutritional status, lifestyle, and mental health among women with PCOS compared to normal in Palestine.

According to our result, lifestyle modification (including physical activity and diet) is the first-line therapy for women with PCOS since lifestyle factors can reduce insulin resistance, improve metabolism and reproductive function, and reduce visceral fat [26]. We compared dietary and physical activity behaviors in women with and without PCOS using a well-defined cohort per the most updated diagnostic criteria available. Our data are consistent with the conclusion that women with PCOS consume similar diets and engage in comparable levels of physical activity compared to women without PCOS. A lack of differences in dietary intake is consistent with previous reports of comparable energy and macronutrient intakes between women with and without PCOS [27–29]. Despite finding a significant relationship between PCOS cases and change in weight and taking any kind of vitamins and supplements, those results were similar to a meta-analysis that found that 67 women with PCOS had a higher rate of weight gain compared to the women without PCOS [30].

The reason why women with PCOS are obese or gain weight may be due to variants in genes such as FTO that play an important role in the determination of body fat mass, and the association between PCOS and obesity is mediated through the effects of the latter on insulin resistance [31].

Unlike others, we didn’t find a significant relationship between smoking and PCOS, which wasn’t consistent with a study conducted in China that provided evidence to support a potential causal association between smoking initiation and an increased risk of PCOS among participants [32].

We also found that there was a direct association between adherence to MD and PCOS, which could support the therapeutic role of single foods and nutrients in the Mediterranean dietary pattern in PCOS. This was similar to a study conducted to study the adherence to the mediterranean diet, dietary patterns, and body composition in women with polycystic ovary syndrome [33], as multiple studies show the role of the Mediterranean diet in decreasing adiposity [34], insulin resistance [35], type 2 DM, and cardiovascular disease [36].

Based on our study, we found that 34.5% of PCOS women needed intervention to get pregnant, and there was a significant relationship between delaying pregnancies and having PCOS. Those results were similar to a study that showed that PCOS is the most common cause of anovulatory infertility, with approximately 90–95% of anovulatory women seeking infertility treatment [37]. However, we didn’t find a significant relationship between pregnancy loss and PCOS, which wasn’t consistent with studies that showed first-trimester miscarriage occurs in 30 to 50% of PCOS women compared with 10 to 15% of normal women [38].

Regarding the premenstrual syndrome, our results pointed out that there is a significant association between PCOS cases and premenstrual syndrome (PCOS cases have severe PMS symptoms in all domains of psychological, physical, and behavioral, *p* = 0.002, 0.000, 0.008, respectively). However, we didn’t find any studies that confirmed or denied our findings. However, this study supports the hypothesis that prolonged anovulation or oligo-ovulation is a symptom of PCOS in women, leading to a distinct deficiency in progesterone and its derivatives. In addition, it was hypothesized that PMS is caused by the deficiency of progesterone and its derivatives, and the PMS symptoms may worsen if there is a progesterone shortage, which may explain why individuals with PCOS experience more severe PMS symptoms [18]. It is worth noting the similarity between premenstrual syndrome (PMS) and polycystic ovary syndrome (PCOS) symptoms, which can be attributed to their shared hormonal imbalances in both conditions [17]. For instance, during the luteal phase of the menstrual cycle, progesterone levels peak before dropping, coinciding with the onset of PMS symptoms [39]. This could explain why individuals with PCOS may experience symptoms like those seen in PMS. Overall, hormonal disturbances present in both PMS and PCOS provide a physiological basis for the similarity in symptoms between the two conditions. Understanding these hormonal mechanisms is crucial for effectively managing and treating both PMS and POCS.

Regarding mental health, our results pointed out that there is a significant association between PCOS. Those results were similar to a study that showed women with PCOS reported greater psychological disturbances compared with controls, with significantly elevated global indices (GSI, PSDI, and PST), designed to measure the overall psychological distress and the intensity of symptoms [40]. Also, those results were similar to a study conducted in Saudi Arabia, which found that the odds of developing depression(*P* = 0.006), anxiety (*P* = 0.028) and stress (*P* = 0.000) were significantly higher in PCOS cases compared to control participants [41]. Another one, conducted in Southwest China, found the prevalence of anxiety (13.3% vs. 2.0%) and depression (27.5% vs. 3.0%) was higher in patients with PCOS compared to the controls (both *P* < 0.05) [42].

Multivariate analysis revealed that the factors associated with PCOS are increasing GHQ scores, which indicated worsening mental health status. Psychiatric symptoms are often undiagnosed among women with PCOS, which may delay appropriate treatment. A study conducted among PCOS women in Sweden demonstrated that these women had a higher prevalence of depression, anxiety, bipolar disorder, schizophrenia spectrum disorders, personality disorder, and eating disorders [43]. PCOS women often experience several problems, such as acne, hirsutism, obesity, menstrual irregularities, and obesity. These may be the reasons for poor self-esteem and psychological disorders [44]. A meta-analysis has documented that women with PCOS have a three-fold higher risk of experiencing depression as compared with non-PCOS women [45]. Another potential reason for this is an imbalance in estrogen levels. Estrogen has an antipsychotic effect. In PCOS, women experience infrequent ovulation, which results in high levels of estrogen. When they ovulate, estrogen levels will decline rapidly, resulting in an increased risk of psychosis [46].

Adherence to the Mediterranean diet (MD) is associated with a lower risk of PCOS. MD is well-known for its health-promoting properties due to its lower saturated fat, low glycemic index, moderate consumption of animal-derived protein, and higher fiber, vitamins, minerals, and antioxidants. A case-control cross-sectional study conducted among 224 Italian women showed that PCOS women consumed less extra-virgin olive oil, legumes, fish, and nuts but a higher intake of simple carbohydrates and saturated fatty acids [33]. Unhealthy dietary habits, especially consumption of simple sugar, may induce inflammation, promote insulin resistance, and worsen hyperandrogenism among PCOS women. MD emphasized the intake of olive oil, which is rich in unsaturated fatty acids and phenolic compounds such as oleocanthal that possess anti-inflammatory properties and improve insulin sensitivity [47]. Besides that, MD is also rich in vitamin E and oleic acid, which have an anti-inflammatory effect and reduce the risk of PCOS-related cancer. MD is also high in resveratrol, naturally found in berries and grapes, which has the potential to reduce androgen production [48]. The level of testosterone is positively correlated with the intake of sugar, saturated fat, and omega-6 fatty acids and negatively correlated with protein, fiber, omega-3 fatty acids, and monounsaturated fatty acids. MD can improve intestinal permeability and gut microbiota composition due to the fiber that acts as a prebiotic, which can reduce mucosal inflammation [49].

In addition, pre-menstrual syndrome (PMS), particularly physical symptoms such as headaches, breast tenderness, and body aches, was associated with PCOS. PMS develops during the late luteal phase of menstruation and disappears at the end of menstruation. It is caused by the interaction between the ovarian hormone and brain neurotransmitters [50]. A study among 20 PCOS patients found that most of the patients complained of physical problems such as pain, discomfort, acne, bloating, facial hair growth, hair loss, darkened skin, and pain in the lower back. Cramping was the most common pain experienced by the patients, especially before or during menstruation. Similarly, another study conducted among 300 students at the University of Sharjah, UAE, found that 95% of the participants complained of experiencing at least one PMS symptom during menstruation [51].

### Strengths

This study has some strengths, including the comprehensive assessment since it provides a thorough examination of various aspects related to polycystic ovary syndrome (PCOS), including weight changes, lifestyle factors such as sleeping problems, reproductive health, mental well-being, premenstrual syndrome (PMS) severity, and dietary patterns. This comprehensive approach enhances our understanding of the multifaceted impact of PCOS on women’s health. In addition, this study utilized established measures such as the General Health Questionnaire (GHQ) and assessments of PMS severity, which are recognized tools for evaluating mental well-being and menstrual health, respectively. This strengthens the validity and reliability of the findings.

### Limitations

This study employed a cross-sectional design, which limits the ability to establish causal relationships between PCOS and the observed outcomes. Future research utilizing longitudinal designs could provide more insights into the temporal relationships between PCOS and various health outcomes. In addition, this study is self-reported, whereas some data, such as lifestyle factors and dietary patterns, were based on self-reported measures, which may be subject to recall bias and social desirability bias. Utilizing objective measures or incorporating validation methods could improve the accuracy of the data collected.

## Conclusion

The findings of this study indicate that individuals with polycystic ovary syndrome (PCOS) had a greater degree of weight change in comparison to women without the condition. A significant relationship was observed between polycystic ovary syndrome (PCOS) and lifestyle factors, particularly perceived sleeping problems. Additionally, a noteworthy connection was identified between PCOS and reproductive health, including delayed pregnancy. The findings of this study demonstrated a significant correlation between polycystic ovary syndrome (PCOS) and a decline in mental well-being, as evidenced by elevated scores on the General Health Questionnaire (GHQ). In regard to PMS severity, women with PCOS reported higher scores in all domains of PMS symptoms. Regarding dietary patterns, reduced adherence to the Mediterranean diet was reported among PCOS cases. Efforts should be made to enhance the lifestyle of women with Polycystic Ovary Syndrome (PCOS) through the implementation of strategies, including the provision of nutrition education and the establishment of well-organized exercise regimens. Future investigations must undertake the task of deciphering the biomarkers that are intricately linked.

## Data Availability

The datasets used and/or analyzed during the current study are available from the corresponding author upon reasonable request.

## Abbreviations

PCOS: Polycystic ovarian syndrome
PMS: Premenstrual syndrome
BMI: Body mass index
WHR: Waist-hip ratio
MD: Mediterranean diet
